# The Performance of the Ethics Committees in Teaching Hospitals Affiliated With Mashhad University of Medical Sciences

**DOI:** 10.5812/ircmj.12332

**Published:** 2014-04-05

**Authors:** Marziyhe Meraji, Farahnaz Sadoughi, Nahid Ramezan Ghorbani, Azar Nezami

**Affiliations:** 1 Department of Medical Records and Health Information Technology, School of Paramedical Sciences, Mashhad University of Medical Sciences and Health Services, Mashhad, IR Iran; 2 Department of Health Information Management, School of Health Management and Information Sciences, Iran University of Medical Sciences, Tehran, IR Iran; 3 Department of Development and Cooperation of Information and Scientific Publications, Undersecretary for Research and Technology, Ministry of Health and Medical Education, Tehran, IR Iran; 4 Shahid Kamyab Hospital, Mashhad University of Medical Sciences, Mashhad, IR Iran

**Keywords:** Task Performance and Analysis, Ethics Committees, Hospitals, Teaching, Iran

## Abstract

**Background:**

During the last three decades, ethics committees have been formed with a growing trend. These committees have a satisfactory and effective impact on the pattern of patient care and its performance. The medical ethics committee is considered one of the most active committees in hospitals, having the aim of providing necessary approaches for the optimal use of the findings in empirical science and diagnostic treatment and observance of Islamic noble values in performing medical affairs.

**Objectives:**

The aim of this study is to assess the performance of the ethics committees, in the teaching hospitals affiliated with Mashhad University of Medical Sciences, in Mashhad.

**Patients and Methods:**

Subjects of this study include teaching hospitals in Mashhad, affiliated with Mashhad University of Medical Sciences and the tool used in gathering the data was a questionnaire, completed based on the information provided by the proceedings of the meetings held by the ethics committees. Also, for the purpose of gathering the suggestions, specifically on the improvement of the performance, a meeting was held with the representatives from hospitals' ethics committees. During the meeting, work reports were presented and recommendations made, based on those presentations. .

**Results:**

Findings of the present study suggest that all hospitals under study, have an ethics committee, of which 85.7% operate in combination with other committees. The composition of the members of the committees, in 57.1% of the hospitals, was based on the guidelines for hospital evaluation.

**Conclusions:**

For the performance of the medical ethics committees to improve, it is recommended that the hospital administration and evaluation section, hold regular meetings and oblige members to participate more actively.

## 1. Background

Human scientific advances are useful and productive for human beings, only when supported by ethics (1). Therefore, clinging to ethics must be considered as a fundamental basis in doctor-patient relationships (2). Ethics is one of the most important aspects in medical sciences. It is essential for the students and physicians, to pay attention to the ethical principles in teachings and treatments (3-5). The progression of biology and health sciences has led to the expansion of critical thinking, in the field of ethics (6). Medical ethics is a structured path to assist physicians, in identifying and analyzing ethical issues in clinical medicine. Good performance of physicians requires knowledge about ethical issues like informed consent, telling the truth, confidentiality and patients’ rights. Ignoring the ethical relationship between patient and health care providers can cause insoluble problems and ethical tensions (7). It is important to note that in medical ethics, the extent and scope in medical decision making should not only be limited to doctor/patient relationships, but the ethical and Islamic principles and values, regarding health domain, should also be considered in every decision made (8).

In Iran, committees, medical councils and hospitals are created in order to achieve the hospitals objectives, assist in program planning, organize and coordinate hospital activities and create an environment of active participation for all employees. Responsibilities and duties of these committees and councils are specified by a guideline, from the Ministry of Health and Medical Education. These committees constitute and reinforce the strength of hospital management (9) and for this purpose, hospital committees have been created, with the aim of improving performance and solving problems and issues in particular. In every general hospital, the presence of a fundamental committee with the aim of providing improvements, in the field of performance, is essential. Additionally, a number of sub-committees, with continuous and effective activities must be created, with the aim of addressing existing deficits and for system improvements. During the last three decades, clinical ethics committees have been formed with a growing trend. These committees have satisfactory and effective impacts on the pattern of patient care and its performances including forming hospital legal policies, concerning ethical matters and issues, individual counseling on clinical issues and organizing educational training for specialists, regarding medical ethics and is considered to be one of the most active committees in hospitals having the aim of providing necessary approaches, for the optimal use of the findings in empirical science and diagnostic treatment and observance of Islamic noble values in performing medical affairs and lastly, providing grounds for development of trust, between the medical community and the public.

The medical ethics committee is composed of a representative of the relevant university's chancellor, a representative of the relevant hospital chief, a veteran medical staff possessing good moral character, a religious retired doctor, possessing a good reputation, commitment, good morality and a good medical record, chosen by the hospital chief with the consent of the former and finally, two reputable doctors, having religious commitments, good morality and known to possess significant medical skills, chosen also by the hospital chief. The committee's meetings must be held at least every two months and it is the responsibility of the hospital chief, to send the minutes of the meetings to the deputy of drugs and treatment affairs of the relevant university. Aside from evaluating and adopting the decisions pertinent to these reports, which include the opinion of the deputy of drugs and treatment affairs, the university is obligated to record these reports in hospitals and use them for hospitals' evaluations.. The minutes of the committees' meetings, must be filed with the name of the committee on it and kept as a hospital record (10).

## 2. Objectives

Therefore, in this study, assessment of the performance of the medical ethics committees of the training hospitals affiliated with Mashhad University of Medical Sciences, has been considered, in order to ensure the optimal use of medical facilities and accurate observance of the noble Islamic values in medical practice.

## 3. Patients and Methods

This was a cross-sectional study, conducted in 2010, using a qualitative approach.. Subjects of the study include all training hospitals affiliated with Mashhad University of Medical Sciences, of which one hospital was eliminated, because its committee had been established in 2009. Minutes from the meetings of the studying medical ethics committees, were evaluated from March to February, through a census. The tool used in data collection was initially a questionnaire, composed of 31 closed questions, having multiple answers (four options). In order to assess the reliability of the questionnaire that was formulated based on standard guidelines and criteria for the evaluation of public hospitals, approved by the Ministry of Health and Medical Education in 2009, opinions of three experts in this field, were used, a pilot study was conducted, and the questionnaire was finalized.

Variables being assessed in this stage included number of meetings held, number of members present in the meetings, issues presented and the guarantee of implementing the approved resolutions. Afterwards data analysis was performed with the Excel software. Also, for the purpose of gathering the suggestions, specifically on the improvement of the teaching hospitals’ ethics committees' performance, a meeting with all representatives from hospital ethics committees, was held on the 26th of May, 2009 at Mashhad University of Medical Sciences. During the meeting, work reports were presented and the meeting was generally focused on the improvement of the committees' performance. For the purpose of gathering data, two people were in charge of taking notes. Analysis was performed immediately after the meeting and the report was prepared, based on the subject matter, with strict observance of its integrity.

## 4. Results

The hospitals under study, included eight training hospitals, affiliated with Mashhad University of Medical Sciences, of which one hospital was omitted because its ethics committee was established on March 2009. All studied hospitals have medical ethics committee. The only ethics committee, performing independently is the Imam Reza Hospital's, which is entitled ethics committee, while the rest of the hospitals’ ethics committees (85.7%) are functioning as combined committees, entitled ethics committee and religious principles. In all hospitals, members of the committees have been preliminarily familiarized with the essential guidelines and regulations and based their activities on these guidelines. The membership duration was one year in 42.8 % in and two years in 28.5% of the hospitals. In 28.5% of hospitals, there was no definite term of membership defined and the term was based on members' performances and when problems arise, members would be replaced immediately. The average number of individuals attending the meetings was six in 71.5% of hospitals and five in the other 28.5% (Table 1).

**Table 1.** Performance of Medical Ethics Committees and Legal Standards of the Teaching Hospitals in Mashhad in 2009

Hospital Performance	Imam Reza	Ghaem	Omolbanin	Khatamolanbiya	Omid	Dr. Sheikh	Shahid Kamyab
**Interval of holding meetings**	2.5 months	3 months	3 months	2.5 months	12 months	6 months	2 months
**Average number of issues raised in each meeting**	2 issues	4 issues	5 issues	2 issues	4 issues	4 issues	4 issues
**Number of meetings held**	5 meetings	4 meetings	4 meetings	5 meetings	1 meeting	2 meetings	6 meetings
**Responsible person for following up committee’s decisions**	secretary of the meeting (division of work)	secretary of the meeting (division of work)	secretary of the meeting	secretary of the meeting	secretary of the meeting + hospital management	manager of the entire hospital committees	secretary of the meeting
**Duration of notification to members**	None	2 years	None	1 year	2 years	1 year	1 year

Among the secretaries chosen for the committees, 57.1% were nurses, while 14.2% were representatives of the hospital chief and 28.5% were other individuals.

 Medical ethics committee’s meetings were chaired by doctors in 42.8% and by the directors in 28.5% of the hospital, while 14.28% were chaired by hospital managers and 

14.28% were chaired by nurses. In 85.7% of hospitals, the chairperson of the meeting is responsible for following up the proceedings of the meetings. However, there exist a division of labor and every member is required to perform his specific duty (Table 2).

**Table 2.** Composition of the Members of the Ethics Committee and Legal Standards Among the Teaching Hospitals, Mashhad, 2009

Composition of the committee members	Imam Reza	Ghaem	Omolbanin	Khatamolanbiya	Omid	Dr. Sheikh	Shahid Kamyab
**Representative of the university’s chancellor**	-	√	-	-	-	√	-
**Representative of the hospital chief**	√	√	√	√	√	√	√
**Employed physician possessing good moral character**	√	√	√	√	√	√	√
**Pious retired physician**	-	-	-	-	-	-	√
**Two reputable physicians recommended by the hospital chief**	√	√	-	√	√	√	√
**Representative from the university’s adaptation plan committee**	-	√	√	-	-	√	-
**Hospital chief’s representative from the staff **		√	-	√	√	√	√
**Representative from the hospital’s Islamic council**		√	-	-	√	√	√
**Religious leaders **		√	-	-	-	√	√
**Female physicians staff recommended by the hospital chief**		√	√	√	√	√	√
**Employed male physicians recommended by the hospital chief**		√		√	√	√	√
**Staff nurse recommended by the hospital’s chief nurse**		√	√	√	√	√	√
**Secretary of the meeting **	BS in midwifery	BS in medical technology	BS in nursing	head nurse	head nurse	head of custody department	head nurse

Topics discussed in meetings were regarding the duties defined for the hospital ethics committee and religious standards in 71.4% of cases and in each session an average number of 4-5 issues, regarding the duties of the ethics committees would be discussed. The findings of the present study, attest to the fact that topics discussed in 85.7% of the medical ethics committees in hospitals under study, were intended to promote moral virtues (based on criteria like respecting patient, privacy and adherence to not committing jobs against the law and recommending to do good), while in 42.8% of the hospitals under study, ethical values were being promoted, through the selection of an individual as a model of ethical behavior. Also, in 14.28 % of hospitals under study, workshops has been conducted, while 85.7% of hospital have focused their activities on distribution of brochures and pamphlets. From chairmen of the committees, 57.14% believed that activities of the medical ethics committees showed positive feedbacks to some extent. Hospital authorities, due to their full participation, have complete knowledge regarding the minutes of the meetings. Also, after each meeting a copy of the decisions made will be sent to all authorities, with complete observance of the ethical rights.

Based on the data analysis of the panel of expert meetings, the main themes extracted from the data were divided into three main groups namely: lack of sufficient recognition of the committee performance, lack of appropriate feedback and failure to implement the important and effective factors in the process of the hospital ethics committees’ performance. Several participants believed that the absence of regular meetings and the inactive participation of the members during the meetings, can be categorized under inaccurate acculturation and lack of appropriate feedback. In this regard, one of the participants expressed that many specialists believe that considering their practice experience and the oath they have taken, majority of the doctors have already been considerate of the ethical issues in their practice, which means establishing an ethics committee will not play an effective role in their performance and these meetings are of little importance to them. Failure to organize courses on capacity building have been expressed by the majority of the participants; they believed that holding training courses and utilization of the experiences of retired doctors in the ethics committee, would not only improve the committee’s performance but would also result in increasing awareness and knowledge of the specialists.

Revision of the duties of the ethics committees' members, promulgated by the Ministry of Health and Medical Education, was also expressed by the majority of the participants in this study; they believed that creating structures and a department entitled “Medical Ethics Committee”, budget allocation and a flat form of ethical policies, would yield positive results. The ministry of health must reform the committees’ structure and must compel each hospital’s medical ethics committee to hold regular monthly meetings.

## 5. Discussion

Assessment of the results of this study, showed that all hospitals under study have medical ethics committees. A study conducted by Gaudine and associates, entitled "Evolution of Hospital Clinical Ethics Committees in Canada (2008)" showed that the percentage of hospitals having medical ethics committee have increased from 58.18% in the years 1984 and 1989 to 85% in 2008 (11). Also, in an study conducted by Ralph Pinnock, Jan Crosthwaite in New Zealand entitled “The Auckland Hospital Ethics Committee”, from the 23 hospitals under study, three had ethics committee, the ethics committee in two hospitals were on the process of creation and the rest of the hospitals, used other committees to solve their ethical dilemmas (12). Another study conducted by Ellen Csikai entitled "The Status of Hospital Ethics Committees in Pennsylvania", has also showed similar results; out of the 208 hospitals surveyed, 183 hospitals (88%) had medical ethics committees (13). In the present study, from 11 teaching hospitals affiliated with the Mashhad University of Medical Sciences, only eight hospitals are studied and the results showed the increasing role of medical ethics in these hospitals.

The study conducted in Australia by Kerridge and associates, entitled “Determining the Function of a Hospital Clinical Ethics Committee”, showed that the most important function of the medical ethics committee is on training and advancement in strategy and policy making and the role of the committee as a counselor has been less supported (14). Also, the study conducted by McGee and Fister on this subject matter in Croatia entitled “Determining the Performance of the Medical Ethics Committee” (2005) showed that regardless of other important functions of the hospital ethics committee, the most important function lies on the analysis of research protocols (7). Assessing the results of the present study, the role and the performance of the medical ethics committees and the problems stated during the committees’ meetings, showed that meetings focused on providing patients’ welfare, observance of personnel’s ethical consideration and training in the field of medical ethics. In another study conducted in the United States (2006), it was shown that 49% of doctors and 58% of nurses believed that medical ethics committee, just like any other committee, must be involved in matters on clinical counseling (15). Also, throughout the investigation conducted by Ralph Pinnock and Crosthwaite in New Zealand in 2004, it was demonstrated that 82% of physicians and 98% of nurses believed that medical ethics advisory group played an important role in resolving ethical problems in clinical practice. In the same research, considering the fact that 68% of physicians and 60% of nurses have acknowledged the role of medical ethics committee, regrettably only 10% of physicians and 6% of nurses have made use of the committee for counseling purposes (12). The study conducted by Ellen L. Csikai and colleagues, entitled “The Status of Hospital Ethics Committees in Pennsylvania” (1998), demonstrated that from the three major functions of the committee (educational training, creating policies and counseling), determining policies have played a more vital role in the performance of these committees (13). All mentioned results and the result of the present study have stressed on the activities of the medical ethics committees in the following fields: developing ethical policies, counseling and educational training on medical ethics.

Results of the present study showed that an average of 64% meetings has been held in 2009 with two months and three months interval, while in the study conducted by Ellen L. Csikai and colleagues entitled “The Status of Hospital Ethics Committees in Pennsylvania” (1998) it was shown that majority of meetings undertaken in the medical ethics committee has taken place in the following manner: 53% monthly, 20% every three months and 14% every two months (13). Also, the study conducted by Gaudine and associates (2010) showed that in the series of meetings held in medical ethics committee, meetings have been conducted in a more regular and accurate manner (11). Results of our study, showed that percentage of attending the meetings for different members of the committee, is as follows: representatives from the university’s chancellors (28.6%), devout retired physicians (14.28%), two reputable physicians nominated by the concerned chancellor (85.7%), representative from the Committee for Adaptation Program (50%), representatives from Islamic Clerics’ Council (50%), representatives from the hospital's chief from the office staff (83.3%) representative from the hospital chief, employed physician possessing good moral characteristics, male and female doctors and nurses (100%). Regarding the same matter, another study showed that members of the committee was composed of 60% physicians, 10% managers, 6% nurses, 5% clerics, 2% social workers and 17% other individuals. Also, the study conducted by Mcgee and Fister showed that the structure and composition of the hospital ethics committees are pursuant to the legal requirements (7). Results of the present study showed that in general, the level of education of the members of the hospitals’ medical ethics committees was as follows: 52% physicians, 9% master degree, 32% bachelor of science and 7% two year diploma course. The study conducted by Burnet et al. (2006) in the United States, concluded that staff and members of the medical ethics committee should have a high knowledge in the field of ethics (15). Results of the present study showed 57.14 % positive feedbacks for the activities implemented by the hospitals' medical ethics committees. The study conducted by Burnet et al. (2006) showed that some physicians failed to refer to this committee due to the following reasons: fear, loss of control, abuse of patient-doctor relationships and the lack of knowledge regarding the medical ethics committee (15).

Finally, due to the reasons that at present accurate assessment on the performance of the medical ethics committee in relation to the manner of holding meetings, composition of the members, activities of the committee and issues to be raised, following up decisions and finally promoting moral virtues cannot be availed, results of this present study showed that the performance of the medical ethics committee on issues like following up of the decisions made and the promotion of moral virtues have gained satisfactory results but in terms of the number of meetings implemented, the presence of members in every meeting and the intervals between these meetings, have only gained average ratings. Therefore, in order to improve the performance of the medical ethics committees, the following recommendations are suggested:

- Evaluation of the performance of the committee in holding regular meetings and controlling the active presence of members by the hospital administration and evaluation office.
- Review of the description of the official duties declared by the Ministry of Health and Medical Education for these committees.
- Emphasizing on the role of the medical ethics committee in the field of educational training on medical ethics, develop ethical policies and provision of ethical counseling.
- The committees’ meetings should be held based on the number of hospital beds and the total number of admissions.
- The presence of a representative from the university in each medical ethics committee’ meeting.
- Utilization of an experienced retired physician as a member of this committee.
- Holding training courses on medical ethics for the committees’ members.
- Hold a more orderly regular pattern for having meetings.
